# PLCγ1 inhibition combined with inhibition of apoptosis and necroptosis increases cartilage matrix synthesis in IL‐1β‐treated rat chondrocytes

**DOI:** 10.1002/2211-5463.13064

**Published:** 2020-12-31

**Authors:** Xiaolei Chen, Ri Chen, Yang Xu, Chun Xia

**Affiliations:** ^1^ Zhongshan Hospital Xiamen University China; ^2^ School of Medicine Xiamen University China

**Keywords:** apoptosis, necroptosis, osteoarthritis, PLCγ1, U73122

## Abstract

Osteoarthritis (OA) is an age‐related, chronic degenerative disease. With the increasing median age of the population, this disease has become an important public health problem. New, disease‐modifying therapies are needed. A potential novel molecular target is phospholipase Cγ1 (PLCγ1), a critical enzyme with important functions including calcium signaling regulation and cell proliferation. In rat chondrocytes treated with IL‐1β (20 ng·mL^−1^ for 36 h), inhibition of PLCγ1 with U73122 (2 μm for 12 h) increased levels and expression of the cartilage matrix components Collagen2 and Aggrecan. This beneficial effect of PLCγ1 inhibition was counteracted by increased chondrocyte apoptosis and necroptosis, increased cell death, and increase levels of ROS, all potentially negative for OA. Combined treatment of IL‐1β + U73122‐treated chondrocytes with inhibitors of apoptosis (Z‐VAD, 10 μm) and necroptosis (Nec‐1, 30 μm) enhanced the increases in levels and expression of Collagen2 and Aggrecan, and prevented the increases in cell death and ROS levels. These results suggest that PLCγ1 inhibition may be a viable approach for an OA therapy, if combined with targeted inhibition of chondrocyte apoptosis and necroptosis.

## Graphical abstract

We found that inhibition of PLCγ1 phosphorylation by U73122 improved cartilage matrix synthesis in IL‐1β‐treated rat chondrocytes. However, it also increased cell apoptosis and necroptosis, which reduced the effect on cartilage matrix synthesis. Inhibition of programmed cell death by simultaneous treatment with apoptosis and necroptosis inhibitors increased chondrocyte proliferation and further enhanced cartilage matrix synthesis.

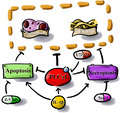

**Abbreviations**
Nec‐1: necrostatin‐1
OA: osteoarthritis
PLCγ1: phospholipase Cγ1
ROS: reactive oxide species
RT‐PCR/qPCR: real‐time quantitative polymerase chain reaction
Z‐VAD: Z‐VAD‐FMK

Osteoarthritis (OA) is a chronic disease that is manifest mainly by cartilage damage and degeneration. It is caused by many factors, but the number one risk factor is age. Worldwide, more than 10% of men and 18% of women over the age of 60 suffer from osteoarthritis [1]. With the increasing median age of the population, the number of OA patients is rising. OA represents a huge medical burden to patients, to their families, and to society [2].

Originally, OA was thought to be caused simply by mechanical wear of articular cartilage [3], but a deeper understanding of the disease shows it to involve lesions of all the joint components and even systemic responses [4]. Despite this, regulation of and changes in the articular cartilage are still the main focus for OA research [5]. Articular cartilage is mainly made up of chondrocytes and a 3D cartilage matrix that is synthesized by the chondrocytes [6]. The cartilage matrix is composed of type II collagen (Collagen2), hyaluronic acid, and proteoglycans or Aggrecans [7, 8]. Changes in the activity of the chondrocytes, or in the levels of the Collagen2 and Aggrecans can lead to changes in the structure and health of the cartilage. These changes may be used as criteria to help quantify OA severity [9].

Treatment of OA includes both surgical and nonsurgical (including pharmaceutical) approaches [10]. With developments in artificial joint replacement, surgical treatment has become the most effective treatment for advanced OA [11, 12]. In comparison, development of nonsurgical and pharmaceutical treatments is relatively limited. Nonsurgical treatments are not as effective as surgical. Pharmaceutical treatments are limited mainly to analgesics, with no effective disease‐modifying drugs currently available [10]. However, artificial joint replacement is not the perfect solution for OA and is associated with problems including joint prosthesis aging and loosening [13, 14]. It is important therefore to continue to search for effective disease‐modifying pharmaceutical treatments for OA focusing on new molecular targets, with the goal of slowing disease progression and preventing or delaying the need for joint replacement. Our focus is on approaches to improve chondrocyte function and survival and thus preserve cartilage matrix synthesis.

Phospholipase Cγ1 (PLCγ1) is a critical enzyme on the cytoplasmic membrane that is involved in transmembrane calcium flux. It can be activated by receptor tyrosine kinase, and its phosphorylation site Tyr783 is the site for the PLCγ1 main function, hydrolysis of PIP2 into DAG and IP3 [17], which regulate Ca^2+^ flow [18]. Several extracellular factors work via PLCγ1 to regulate cell proliferation, differentiation, migration, apoptosis, and autophagy [15, 16]. U73122 is a commonly used PLCγ1 inhibitor and can effectively inhibit IP3 synthesis and calcium release [19]. A previous study showed that U73122 intra‐articular injection in rats reduced OA damage [20]. However, there are also studies showing that U73122 increases cell apoptosis [21, 22].

Apoptosis was the first described form of programmed cell death. It can involve caspase activation and is accompanied by pyknosis, karyolysis, and apoptotic body formation [23, 24]. Necroptosis is another form of programmed cell death and is different from classic necrosis. In necroptosis, receptor‐interacting protein 1/3 (RIP1/3) activates a series of death signal systems, which can destroy organelles such as mitochondria and lead to cell death [25, 26]. Previous studies have shown that both apoptosis and necroptosis in chondrocytes are OA risk factors [27, 28, 29, 30].

In order to explore new ideas for pharmaceutical treatment of OA, we used Il‐1β‐activated chondrocytes to mimic the OA state and investigated the effects of inhibiting PLCγ1 (with U73122) [20], apoptosis (with caspase inhibitor Z‐VAD‐FMK or Z‐VAD), and necroptosis (with RIP1 inhibitor necrostatin‐1 or Nec‐1), alone and in combination, on production of the cartilage matrix components Collagen2 and Aggrecan.

## Materials and methods

### Antibodies and reagents

Antibodies used in this study were purchased from the following companies: antibodies against Collagen2 (1 : 1000), Bcl‐2 (1 : 1000), Bax (S757) (1 : 1000), P53 (1 : 1000), RIP1 (1 : 1000), and RIP3 (1 : 1000) were purchased from Abcam Inc. (Cambridge, MA, USA); antibodies targeting PLCγ1 (1 : 1000), p‐PLCγ1 (Y783) (1 : 1000), and caspase3 (1 : 1000) were purchased from Cell Signaling Technology Inc. (Beverly, MA, USA); and antibodies against Aggrecan (1 : 1000) and β‐actin (1 : 40 000) were purchased from Sigma‐Aldrich in China (Shanghai, China), respectively. Inhibitors used in this study (U73122, Z‐VAD, and Nec‐1) were purchased from MedChemExpress (Monmouth Junction, NJ, USA). Cytokine used in this study such as recombinant rat IL‐1β was purchased from PeproTech (Rocky Hill, NJ, USA). Other reagents were of the highest grade commercially available.

### Isolation, culture, and treatment of rat chondrocytes

All the operations were approved by the committee on the Ethics of Animal Experiments of Xiamen University (ID no. 20170301).

Chondrocytes were isolated from knee cartilage of neonatal Sprague Dawley rats (within 1–2 days after birth) by mechanical and collagenase digestions [31, 32]. Primary chondrocytes were cultured in DMEM/F12 with 10% fetal bovine serum and penicillin (100 U·mL^−1^)/streptomycin (0.1 mg·mL^−1^). At 80% confluence, the cells were then plated in 60‐mm cell culture dishes at 1 : 3 or 1 : 4 at 37 °C, 95% humidity, and 5% CO_2_, and F2 generation cells were used for the experiments. IL‐1β (10, 20, and 40 ng·mL^−1^) when studied alone was added to the culture for 36 h to activate the cells [32]. The inhibitors U73122 (1, 2, 4, and 6 μm), Z‐VAD (10 μm), and Nec‐1 (30 μm) were added to the IL‐1β‐stimulated chondrocyte cell culture for 12 h. Those inhibitors were added for the last 12 h of the 36 h of IL‐1β. When studied together, inhibitors were added at 10‐min intervals.

### Western blotting analysis

Protein extracts were subjected to SDS‐PAGE (8–15%) and transferred to a PVDF membrane (GE Healthcare, Hertfordshire, UK) as described before [33, 34]. Briefly, the PVDF membrane was clipped and incubated with the above‐mentioned primary antibodies at 4 °C overnight, followed by complete elution of the primary antibodies and the addition of the corresponding secondary antibodies at room temperature for 1 h. An enhanced chemiluminescence (ECL) detection kit was used to detect antibody reactivity (Pierce, Rockford, IL, USA).

### Cell viability assay

For the cell viability assay, 1 × 10^3^ cells were cultured in 96‐well plates and were treated with IL‐1β and the inhibitors as described. At the end of the treatment period, 3‐(4,‐dimethylthiazol‐2‐y)‐2, 5‐diphenyl‐tetrazolium bromide (MTT reagent) was added to the cultures as per the manufacturer’s instruction and as described in a previous study [35]. After 4 h, DMSO was added to stop the reaction and solubilize the formazan. The optical density was measured at 490 nm with GloMax 20/20 luminometer (Promega, Madison, WI, USA).

### Real‐time quantitative polymerase chain reaction (RT‐PCR/qPCR)

Total RNA was extracted from the chondrocytes using TRIzol (Invitrogen, Carlsbad, CA, USA), and cDNA was synthesized with 1 μg of total RNA at 37 °C for 15 min using a PrimeScript RT Master Mix Kit (Takara, Dalian, China). Real‐time PCR was then performed using a Roche LightCycler 96 (Roche, Basel, Switzerland) with a SYBR Premix Ex Taq II Kit (Takara). The results were normalized to GAPDH and analyzed using sds software v2.1 as previously described [33, 34]. The following primers were used in quantitative PCR for measuring gene expression relative to GAPDH.

Primer sequences used in this study were as follows:

Collagen2:
  Forward 5‐TCCTAAGGGTGCCAATGGTGA‐3,
  Reverse 5‐GGACCAACTTTGCCTTGAGGAC‐3;
Aggrecan:
  Forward 5‐TCCGCTGGTCTGATGGACAC‐3,
  Reverse 5‐CCAGATCATCACTACGCAGTCCTC‐3;
GAPDH:
  Forward 5‐CAAGTTCAACGGCACAGTCAAG‐3,
  Reverse 5‐ACATACTCAGCACCAGCATCAC‐3.

### Apoptosis and necroptosis analysis

The detection of apoptotic cells was performed using a Beckman CytoFlex (Beckman, Brea, CA, USA) with the Annexin V‐FITC/PI detection kit (Beyotime Biotechnology, Shanghai, China), as per the manufacturer's instructions. The results were analyzed using cytexpert 1.2.11.0 and flowjo 10 as previously described. The percentage of cell death (including dying and dead cells) was calculated from the total number of viable apoptotic cells and nonviable apoptotic cells (from Annexin V‐FITC with and without PI staining) [36]. Protein levels of apoptosis and necroptosis indices Bcl‐2, Bax, P53, pro/cleaved‐caspase3, RIP1, and RIP3 were analyzed by Western blotting.

### Reactive oxide species (ROS) analysis

ROS were analyzed with the ROS detection kit (Beyotime Biotechnology), as per the manufacturer's instructions. The results were analyzed using cytexpert 1.2.11.0 and flowjo 10 as previously described [37].

### Statistical analysis

Data are expressed as the mean ± 95% confidence interval (CI) of three independent experiments for each experiment. One‐way analysis of variance (ANOVA) with the Dunnett test was used to compare the control group with treatment groups by graphpad prism 5 software (GraphPad Software, San Diego, CA, USA). Differences at a value of *P* < 0.05 were regarded as statistically significant.

## Results

### Effects of PLCγ1 inhibitor U73122 on Collagen2 and Aggrecan levels in IL‐1β‐treated rat chondrocytes

**Fig. 1:**
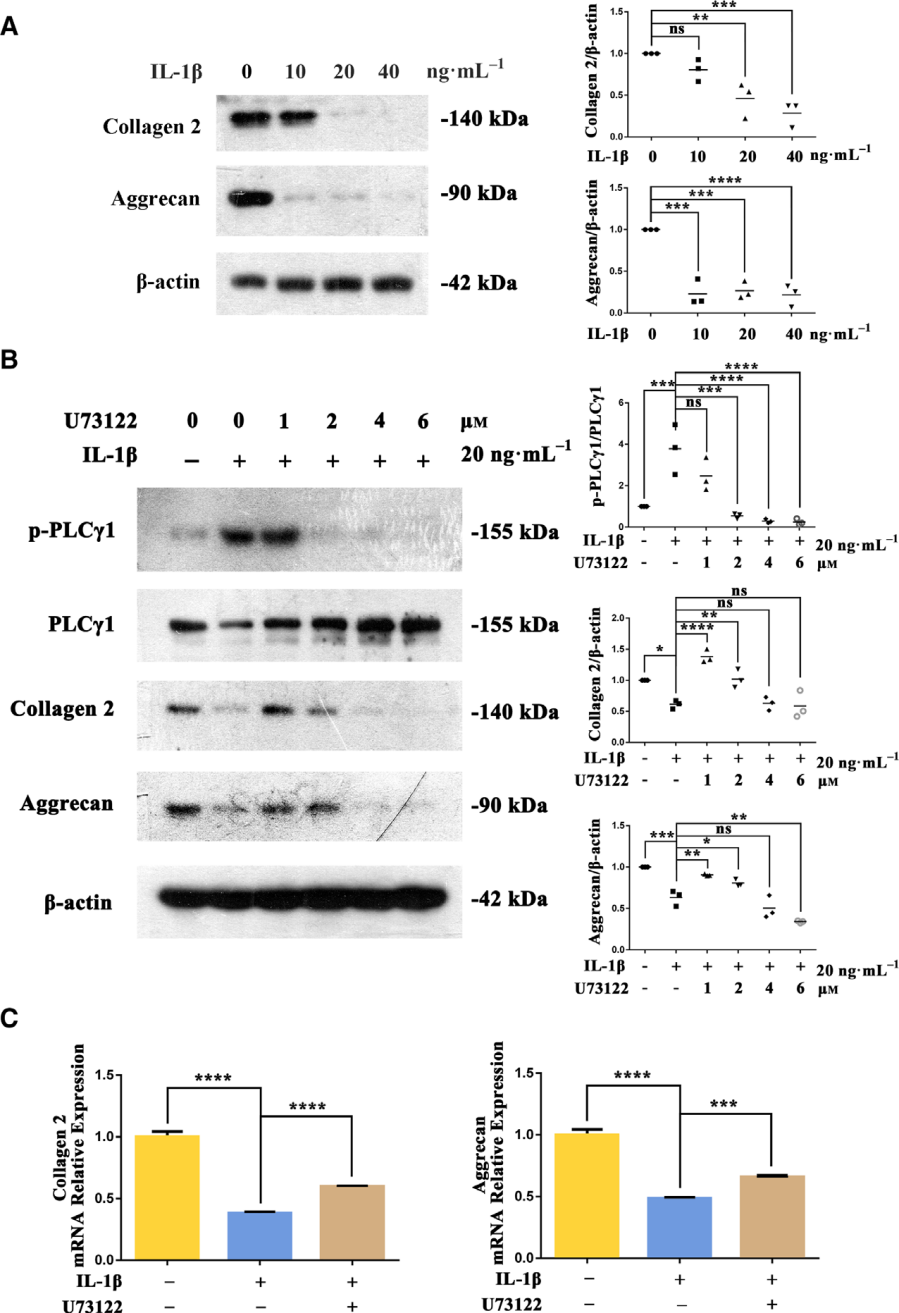
Inhibition of PLCγ1 with U73122 increased Collagen2 and Aggrecan levels in IL‐1β‐treated rat chondrocytes. Rat chondrocytes were treated with IL‐1β at 10, 20, and 40 ng·mL^−1^ for 36 h. Protein levels of Collagen2 and Aggrecan were analyzed by Western blotting (A). Chondrocytes pretreated with IL‐1β (20 ng·mL^−1^ for 36 h) were treated with the PLCγ1 inhibitor U73122 at 1, 2, 4, and 6 μm for 12 h. Total and phosphorylated protein levels of PLCγ1, Collagen2, and Aggrecan were analyzed by Western blotting (B). In addition, chondrocytes pretreated with IL‐1β (20 ng·mL^−1^ for 36 h) were treated with U73122 (2 μm for 12 h), and mRNA levels of Collagen2 and Aggrecan were analyzed by RT‐PCR (C). β‐Actin was used as the control for Western blotting, and GAPDH was used as the control for RT‐PCR. Values are means and standard deviations, the error bars represent SD. One‐way ANOVA with the Dunnett test was used to calculate *P* values. These results are representative of at least three independent experiments in each experiment. *P < 0.05, **P < 0.01, ***P < 0.001, ****P < 0.0001.

To mimic the OA state, rat chondrocytes were treated with different concentrations of IL‐1β (10, 20, and 40 ng·mL^−1^) for 36 h. Collagen2 and Aggrecan levels decreased significantly at IL‐1β 20 and 40 ng·mL^−1^ (Fig. 1A). To determine effects of PLCγ1 inhibition, chondrocytes treated with IL‐1β (20 ng·mL^−1^) were treated with the PLCγ1 inhibitor U73122 at 1, 2, 4, and 6 μm for 12 h. Compared with the IL‐1β‐treated control group, U73122 at 2 μm significantly decreased the phosphorylation of PLCγ1 (Figs. 1B and S1). This was accompanied by significantly higher Collagen2 and Aggrecan protein levels (Figs. 1B and S1) and mRNA levels (Fig. 1C).

Higher concentrations of U73122 (4 and 6 μm) also significantly reduced PLCγ1 phosphorylation but did not increase Collagen2 and Aggrecan levels compared with the IL‐1β‐treated control group (the Aggrecan level at 6 μm was significantly decreased). Interestingly, compared with the 1 μm group, U73122 at 2 μm had a significantly greater decrease in PLCγ1 phosphorylation but a smaller increase in Collagen2 and Aggrecan levels.

### Effects of U73122 alone and combined with Z‐VAD and Nec‐1 on chondrocyte apoptosis and necroptosis

**Fig. 2:**
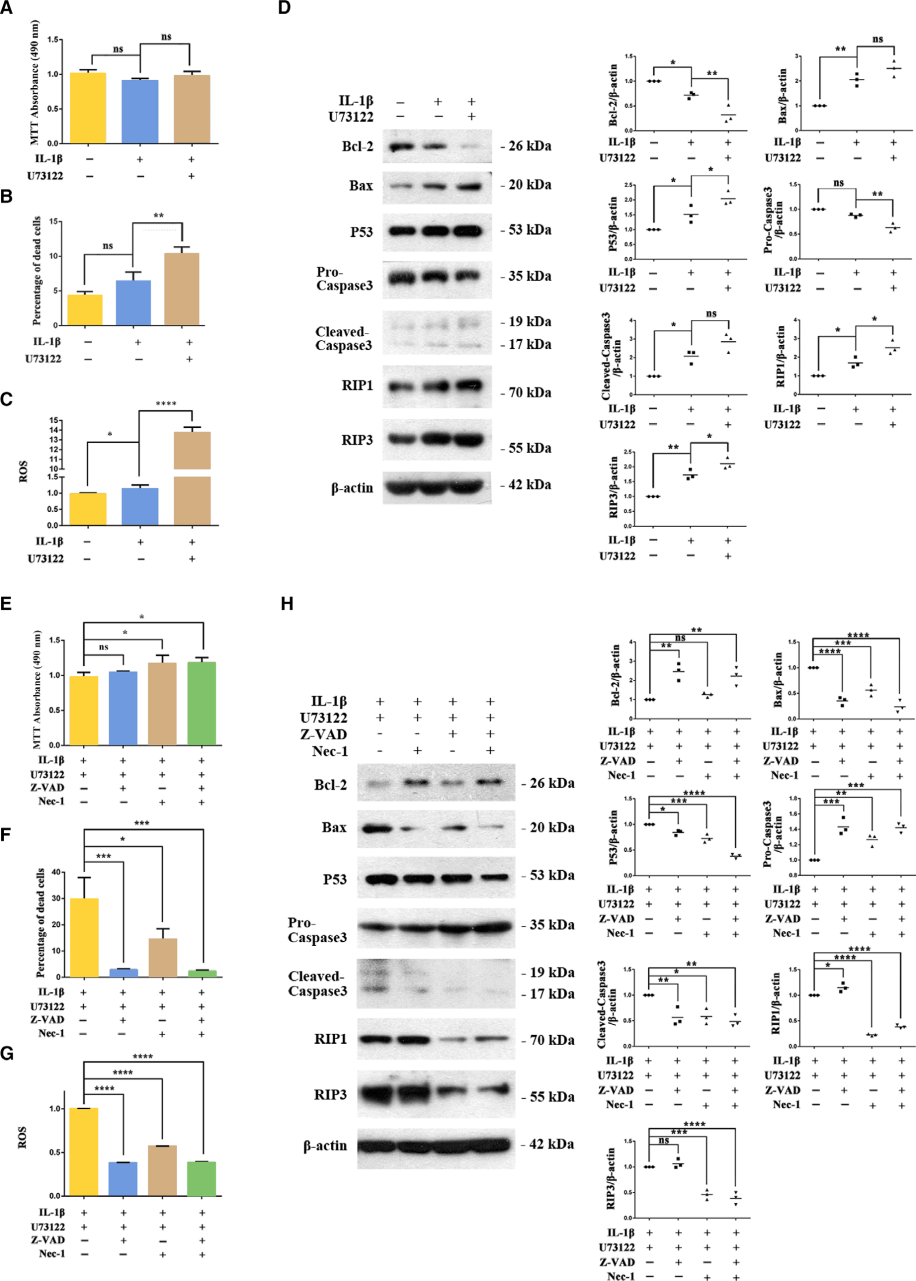
U73122‐induced apoptosis and necroptosis in IL‐1β‐treated rat chondrocytes, and the effect of combined treatment with the apoptosis and necroptosis inhibitors Z‐VAD and Nec‐1, respectively. Rat chondrocytes pretreated with IL‐1β (20 ng·mL^−1^ for 36 h) were treated with U73122 (2 μm for 12 h). Cell proliferation (A), percentage of dead cells (B), and ROS level (C) were measured. Protein levels of apoptosis and necroptosis indexes Bcl‐2, Bax, P53, pro/cleaved‐caspase3, RIP1, and RIP3 were analyzed by Western blotting (D). Chondrocytes pretreated by IL‐1β (20 ng·mL^−1^ for 36 h) and U73122 (2 μm for 12 h) were treated with Z‐VAD (10 μm for 12 h) or/and Nec‐1 (30 μm for 12 h). Cell proliferation (E), percentage of dead cells (F), and ROS level (G) were measured, and protein levels of apoptosis and necroptosis indexes were analyzed by Western blotting (H). The results of ROS were normalized, means of the first group in Fig. 2C,G were used as the control for ROS. β‐Actin was used as the control for Western blotting. Values are means and standard deviations, the error bars represent SD. One‐way ANOVA with the Dunnett test was used to calculate *P* values. These results are representative of at least three independent experiments in each experiment. *P < 0.05, **P < 0.01, ***P < 0.001, ****P < 0.0001.

The MTT assay showed that IL‐1β (20 ng·mL^−1^) did not affect chondrocyte proliferation with or without PLCγ1 inhibition with U73122 (2 μm) (Fig. 2A). However, compared with the IL‐1β‐treated group the percent dead cells increased significantly in the IL‐1β + U73122‐treated group (Annexin V‐FITC/PI assay; Fig. 2B). ROS levels significantly increased in the IL‐1β‐treated group vs untreated control group (Fig. 2C), and levels were further increased significantly in the IL‐1β + U73122 group.

Changes in markers of apoptosis and necroptosis were consistent with IL‐1β‐induced chondrocyte apoptosis and necroptosis that were further increased by PLCγ1 inhibition with U73122 (Fig. 2D). Bcl‐2 level decreased in the IL‐1β group (vs untreated group) and further decreased significantly in the IL‐1β + U73122 group (vs IL‐1β‐treated group). Bax and p53 levels increased in the IL‐1β group (vs untreated group) and further increased significantly in the IL‐1β + U73122 group (vs IL‐1β‐treated group). In the IL‐1β group, there was a small but not significant decrease in the pro‐caspase3 level and a significant increase in the cleaved‐caspase3 level (vs untreated group). In the IL‐1β + U73122 group, the pro‐caspase3 level was further decreased and the cleaved‐caspase3 level showed a further small but not significant increase (vs IL‐1β‐treated group). Levels of the necroptosis markers RIP1 and RIP3 increased in the IL‐1β group (vs untreated group) and were further increased significantly in the IL‐1β + U73122 group (vs IL‐1β‐treated group).

We hypothesized that PLCγ1 inhibition with U73122 could increase Collagen2 and Aggrecan levels, but the effect was offset by increased chondrocyte apoptosis and necroptosis, especially at higher PLCγ1 inhibition. To try to separate these effects, in IL‐1β + U73122‐treated chondrocytes, we inhibited apoptosis (with Z‐VAD) and necroptosis (with Nec‐1) alone and combined. Compared with the IL‐1β + U73122‐treated group, combined treatment with Z‐VAD (10 μm) and Nec‐1 (30 μm) significantly increased chondrocyte proliferation (Fig. 2E), significantly decreased the percentage of dead cells, and significantly decreased ROS levels (Fig. 2F,G). With Z‐VAD treatment alone, changes in markers were consistent with inhibition of apoptosis (Fig. 2H). Compared with the IL‐1β + U73122‐treated control group, the Bcl‐2 level was increased, Bax and P53 levels were decreased, pro‐caspase3 level was increased, and cleaved‐caspase3 level was decreased. There were small significant increases in RIP1 and RIP3 with Z‐VAD treatment alone compared with the control group. With Nec‐1 treatment alone, changes in markers were consistent with inhibition of necroptosis (Fig. 2H). Compared with the IL‐1β + U73122‐treated control group, RIP1 and RIP3 levels were significantly reduced. Treatment with Nec‐1 alone also appeared to reduce apoptosis with decreases in Bax, P53, and cleaved‐caspase3 (vs IL‐1β + U73122‐treated group), but these effects were less than with Z‐VAD treatment. The largest changes in markers of apoptosis and necroptosis were observed in the combined Z‐VAD and Nec‐1‐treated group.

### U73122 combined with apoptosis and necroptosis inhibitors increased Collagen2 and Aggrecan levels in IL‐1β‐treated rat chondrocytes

**Fig. 3:**
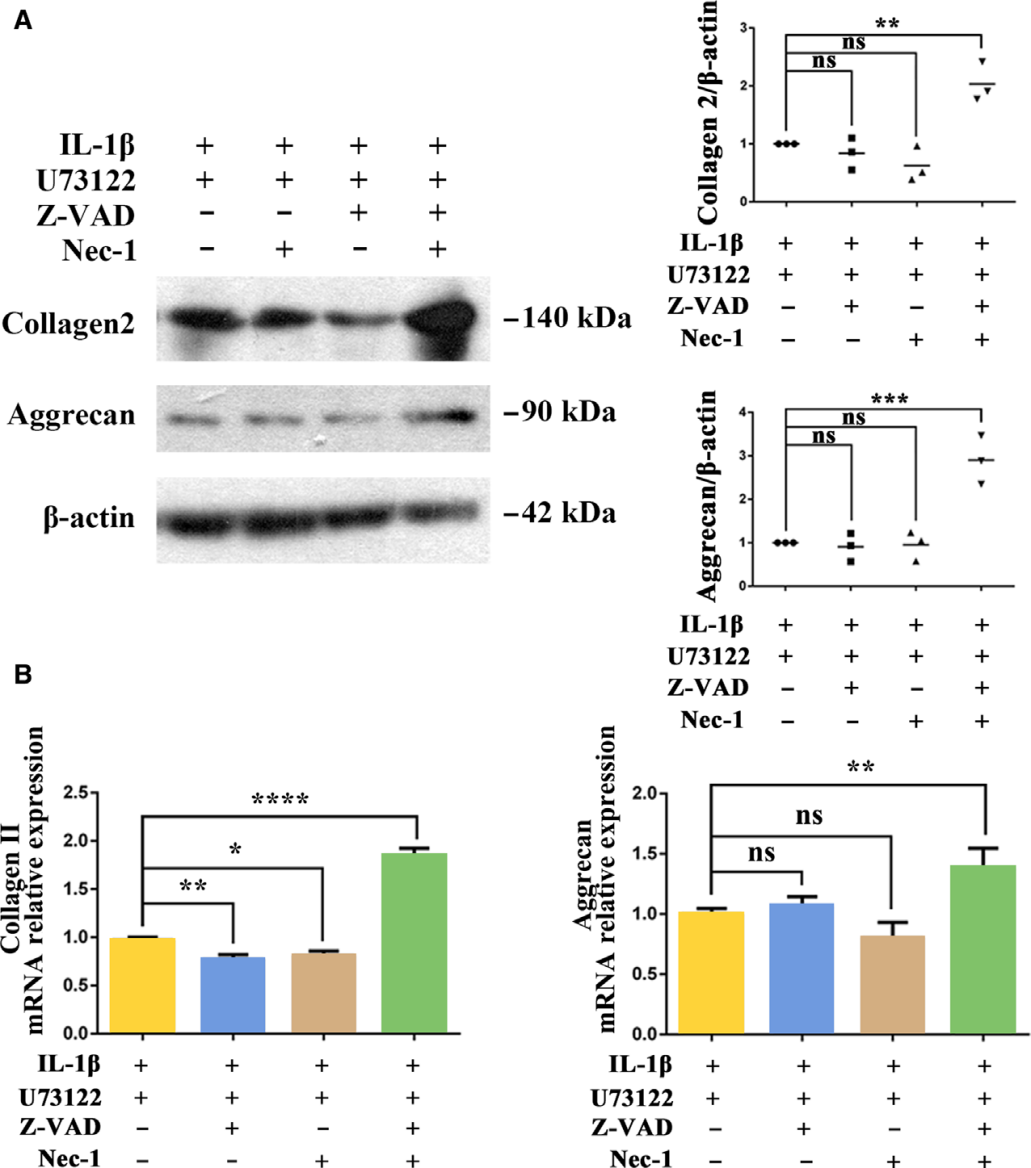
U73122 combined with apoptosis and necroptosis inhibitors increased Collagen2 and Aggrecan levels in IL‐1β‐treated rat chondrocytes. Chondrocytes pretreated by IL‐1β (20 ng·mL^−1^ for 36 h) and U73122 (2 μm for 12 h) were treated with Z‐VAD (10 μm for 12 h) or/and Nec‐1 (30 μm for 12 h). Protein (A) and mRNA (B) levels of Collagen2 and Aggrecan were analyzed by Western blotting and RT‐PCR, respectively. β‐Actin was used as the control for Western blotting, and GAPDH was used as the control for RT‐PCR. Values are means and standard deviations, the error bars represent SD. One‐way ANOVA with the Dunnett test was used to calculate *P* values. These results are representative of at least three independent experiments in each experiment. *P < 0.05, **P < 0.01, ***P < 0.001, ****P < 0.0001.

Treatment with Z‐VAD or Nec‐1 alone had no significant effect on Collagen2 or Aggrecan levels compared with the IL‐1β + U73122‐treated control group (Fig. 3A). Both Z‐VAD and Nec‐1 produced small decreases in Collagen2 mRNA level but had no effect on Aggrecan mRNA level. (Fig. 3B). Combined treatment with Z‐VAD and Nec‐1 significantly increased protein and mRNA levels of Collagen2 and Aggrecan compared with the IL‐1β + U73122‐treated control group.

## Discussion

As one of the most important members of the phospholipase family, the role of PLCγ1 in osteoarthritis, especially in chondrocytes, has become a research focus [38, 39]. In a previous study in a Sprague Dawley rat model of OA, we showed that intra‐articular injection of the PLCγ1 inhibitor U73122 reduced cartilage damage [20]. This suggested that targeted inhibition of PLCγ1 might be a potential therapy for OA. In this current study in IL‐1β‐treated rat chondrocytes, we found that a low concentration (2 μm) of the PLCγ1 inhibitor U73122 decreased PLCγ1 phosphorylation and improved cartilage matrix synthesis, with increased levels of Collagen2 and Aggrecan. However, higher concentrations of U73122 reduced levels of Collagen2 and Aggrecan. These observations are consistent with our previous results [40] and with those of Gao [41], who found U73122 10 μm reduced Collagen2 and Aggrecan mRNA levels in rat nucleus pulposus cells. Our subsequent experiments showed this decreased matrix synthesis to result from increased apoptosis and necroptosis of chondrocytes at the higher concentrations of U73122 and higher level of PLCγ1 inhibition.

The conclusion from these observations is that PLCγ1 inhibition and cartilage matrix synthesis is not a simple linear relationship, and there might be a crossover point between them such that a successful therapy for OA would provide some but not complete PLCγ1 inhibition.

Increased chondrocyte death and ROS levels are risk factors for OA [42], and the results of our experiments are consistent with these factors being associated with reduced cartilage matrix synthesis. Increases in cell death and ROS levels are closely related to programmed cell death (PCD), especially apoptosis and necroptosis, and apoptosis and necrosis inhibitors can reduce both factors [43]. Our current results are also consistent with this. A study by Yuan *et al*. [21] showed that inhibition of PLCγ1 phosphorylation with U73122 10 μm increased apoptosis in pheochromocytoma 12 (PC12) cells induced by hydrogen peroxide. A study by Jiang *et al*. [22] also showed that knockdown of PLCγ1 can increase apoptosis of vascular smooth muscle cells. The increased level of chondrocyte apoptosis after PLCγ1 inhibition with U73122 in our studies was similar to that observed by Xiao *et al*. [39]. In addition, chondrocyte apoptosis is a risk factor for the initiation and development of OA, and inhibition of chondrocyte apoptosis can relieve OA [27, 28]. Our current study results are consistent with these data.

Although necroptosis is currently a focus for research in programmed cell death, the direct relationship between PLCγ1 and necroptosis is still not clear. However, it is clear that PLCγ1 is an important regulator of Ca^2+^, and Chang *et al*. have shown that Ca^2+^ regulates RIP3 through CaMKII, thus affecting the necroptosis level in rat ventricular cardiomyocytes [44, 45]. We therefore had reason to believe there was a relationship between PLCγ1 and necroptosis in chondrocytes. This was confirmed, and our results showed that chondrocytes necroptosis increased with PLCγ1 inhibition with U73122 treatment.

It is worth noting that in this study, Z‐VAD and Nec‐1 treatment alone did reduce chondrocyte apoptosis and necroptosis, respectively, but neither improved cartilage matrix synthesis. Previous reports show that apoptosis and necroptosis share the same signal pathway in the early stage, and it is difficult to completely inhibit programmed cell death by inhibiting just one process [46]. In our study, combined inhibition of apoptosis and necroptosis with Z‐VAD and Nec‐1 combined produced greater inhibition of cell death and increased cartilage matrix synthesis.

The observation of enhanced cartilage matrix synthesis at a low concentration of U73122 but reduced cartilage matrix synthesis at a higher concentration is not rare in pharmaceutical research and clinical drug usage [47, 48, 49]. For example, except for leukemia [50], tretinoin is also used in photodamaged skin and cosmetology [51]. However, because of its strong inhibition of keratinization a large overdose can damage the skin, causing skin erythema and ulceration [52, 53]. Although U73122 at 2 μm improved cartilage matrix synthesis, this concentration did increase chondrocyte apoptosis and necroptosis levels, which would partially counteract the effect on matrix synthesis. The full and potentially curative effect on matrix synthesis was apparent when the combined treatment with Z‐VAC and Nec‐1 was used to inhibit the U73122‐induced increases in apoptosis and necroptosis, respectively. This is similar to the situation of reducing or alleviating the skin erythema and ulceration caused by tretinoin treatment by reducing drug dose or by combining with nonsteroidal drugs [52, 53]. Another example of ‘cocktail therapy’ is the treatment of AIDS, where use of multiple drugs can reduce drug resistance and side effects, and improve the overall therapeutic effect through the synergistic effects of the multiple drugs [54].

**Fig. 4:**
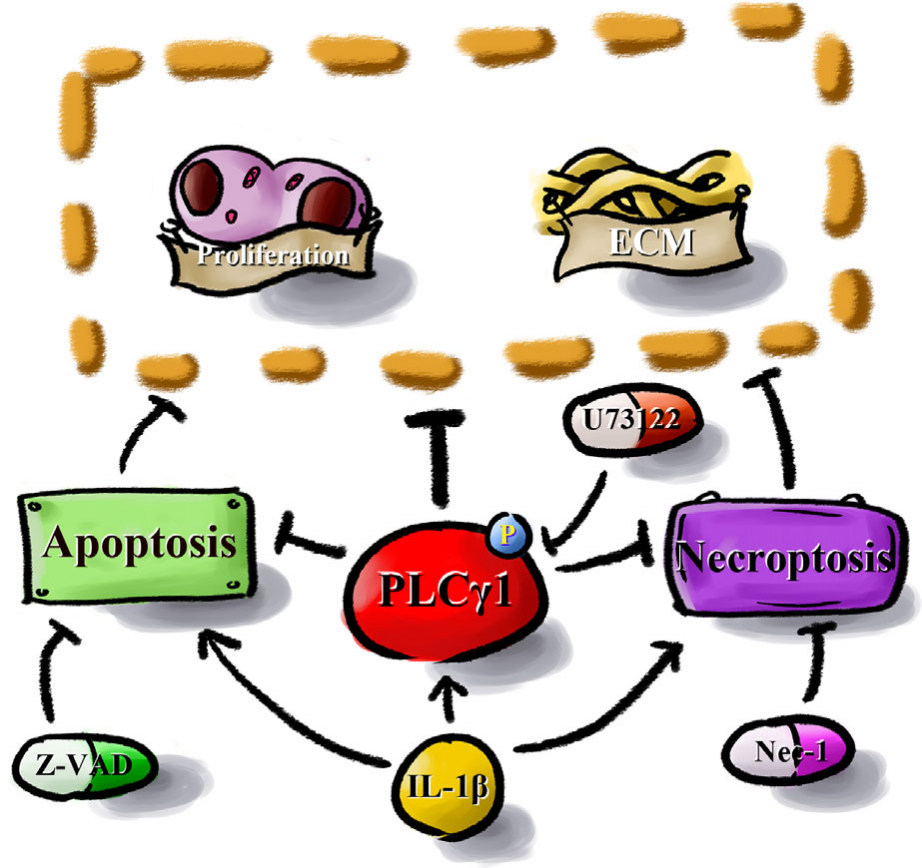
Molecular mechanism of PLCγ1 inhibition combined with inhibition of apoptosis and necroptosis increases cartilage matrix synthesis in IL‐1β‐treated rat chondrocytes. Inhibition of PLCγ1 phosphorylation by U73122 improved cartilage matrix synthesis in IL‐1β‐treated rat chondrocytes. However, it also increased cell apoptosis and necroptosis, which reduced the effect on cartilage matrix synthesis. Inhibition of programmed cell death by simultaneous treatment with the apoptosis and the necroptosis inhibitor increased chondrocyte proliferation and further enhanced cartilage matrix synthesis.

In summary, inhibition of PLCγ1 phosphorylation by U73122 improved cartilage matrix synthesis in IL‐1β‐treated rat chondrocytes. However, it also increased cell apoptosis and necroptosis, which reduced the effect on cartilage matrix synthesis. Inhibition of the U73122‐induced programmed cell death by simultaneous treatment with the apoptosis inhibitor Z‐VAD and the necroptosis inhibitor Nec‐1 increased chondrocyte proliferation and further enhanced cartilage matrix synthesis. Therefore, we propose PLCγ1 as a new molecular target as a potential disease‐modifying therapy for OA (Fig. 4).

## Conflicts of interest

The authors declare no conflict of interest.

## Author contributions

XC and CX conceived the study and designed the experiments. XC, RC, and YX contributed to the data collection. XC and RC performed the data analysis and interpreted the results. XC wrote the manuscript. CX contributed to the critical revision of the article. All authors read and approved the final manuscript.

## Supporting information


**Fig. S1.** U73343 can’t inhibit PLCγ1 and also can’t increase Collagen2 and Aggrecan levels in IL‐1β‐treated rat chondrocytes. Rat chondrocytes pretreated with IL‐1β (20 ng/ml for 36 hours) were treated with U73122 or U73343 (2 μM for 12 hours).

## Data Availability

The data will be available from the corresponding author upon reasonable request.
