# Supplementary figures and images for: PLCγ1 inhibition combined with inhibition of apoptosis and necroptosis increases cartilage matrix synthesis in IL‐1β‐treated rat chondrocytes
Source: FEBS Open Bio. 2020 Dec 31;11(2):435–45. doi: 10.1002/2211-5463.13064 (PMC7876495; doi:10.1002/2211-5463.13064)

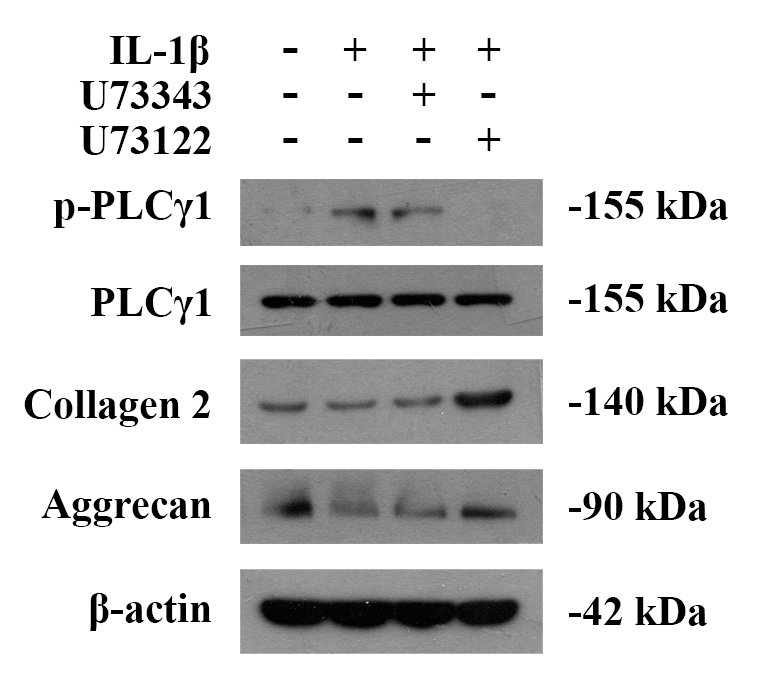

Supplement: Supplementary file 1 — Fig. S1. U73343 can’t inhibit PLCγ1 and also can’t increase Collagen2 and Aggrecan levels in IL‐1β‐treated rat chondrocytes. Rat chondrocytes pretreated with IL‐1β (20 ng/ml for 36 hours) were treated with U73122 or U73343 (2 μM for 12 hours). [file FEB4-11-435-s001.tif]
